# Supplementary material for: Structure Identification of ViceninII Extracted from Dendrobium officinale and the Reversal of TGF-β1-Induced Epithelial–Mesenchymal Transition in Lung Adenocarcinoma Cells through TGF-β/Smad and PI3K/Akt/mTOR Signaling Pathways
Source: Molecules. 2019 Jan 2;24(1):144. doi: 10.3390/molecules24010144 (PMC6337427; doi:10.3390/molecules24010144)
Supplement: Supplementary file 1 [file molecules-24-00144-s001.pdf]

We certify that the following article

Structure Identification of Vicenin Ⅱ Extracted from Dendrobium officinale and It's Effect of Reversing TGF-β1-induced Epithelial-Mesenchymal Transition in Lung Adenocarcinoma Cells through TGF-β/Smad and PI3K/Akt/mTOR Signaling Pathways

Gang Wei

has undergone English language editing by MDPI. The text has been checked for correct use of grammar and common technical terms, and edited to a level suitable for reporting research in a scholarly journal. MDPI uses experienced, native English speaking editors. Full details of the editing service can be found at ► www.mdpi.com/authors/english.

MDPI Multidisciplinary Digital Publishing Institute

Basel, November 2018

Martyn Rittman, Ph.D.
English Editing Manager
englishediting@mdpi.com
http://www.mdpi.com/authors/english
